# The effect of technology use on sleep quality in primary school-aged children

**DOI:** 10.1055/s-0046-1827211

**Published:** 2026-08-31

**Authors:** Feyza Dereli, Merve Ercan

**Affiliations:** 1 Izmir Katip Celebi University, Faculty of Health Sciences, Public Health Nursing Department, Izmir, Turkey.; 2 Izmir Katip Celebi University, Faculty of Health Sciences, Izmir, Turkey.

**Keywords:** Child, Sleep Quality, Information Technology, Smartphone, Schools

## Abstract

**Background:**

Childhood sleep problems are an increasing public health concern. Although screen-based technology use is a potential cause, the specific effects of distinct screen-based behaviors on children's sleep remain unclear.

**Objective:**

To examine associations between technology use characteristics and sleep quality among primary school-aged children.

**Methods:**

The present descriptive and correlational study was conducted based on parent reports from 600 primary school students. Sleep quality was assessed using the Children's Sleep Habits Questionnaire (CSHQ). The following were investigated: sociodemographic variables, television, tablet, and smartphone usage times; the presence of technological devices in the children's rooms; and technology use before bedtime. Group comparisons were made using non-parametric tests, and a multiple linear regression analysis was used to examine the independent predictors of the CSHQ total score.

**Results:**

The mean CSAQ score for children was 52.79 ± 6.73, and 98.7% of participants were above the clinical sleep problem threshold. The utilization of technological devices was found to be associated with a substantial effect on sleep quality. The utilization of technology prior to bedtime, in addition to smartphone usage that exceeds 2 hours per day, has been demonstrated to be significantly associated with elevated CSHQ scores. In the multiple regression analysis, it was identified that smartphone use (> 2 hours/day) was the strongest independent predictor of sleep quality (β = 0.34; *p* < 0.001).

**Conclusion:**

The findings of the present study indicate that smartphone use, particularly before bedtime and for extended periods, has a substantial and adverse effect on the quality of sleep in elementary school children.

## INTRODUCTION

It is imperative to recognize the significance of adequate and quality sleep for the healthy physical, cognitive, and emotional development of children. 1 2 While there is evidence to suggest that sleep duration and sleep habits may vary regionally and culturally, the American Academy of Sleep Medicine (AASM) recommends a minimum of 9 to 12 hours of sleep for school-aged children. 3 4 However, it is reported that approximately 30% of children experience difficulties falling asleep and staying asleep. The phenomenon of uncontrolled and prolonged technology use is widely considered to be a significant risk factor for the development of sleep problems in childhood. 5 6 7

A plethora of studies in the exisitng literature have focused on the effects of problematic smartphone use and screen time on sleep behaviours in children and adolescents. 5 However, these studies are mostly based on adolescent samples or examine the relationship between screen time and sleep duration in early childhood. 7

Furthermore, previous research investigating the relationship between technology use and sleep quality in children has generally focused on single devices or limited behavioral variables. Research employing multidimensional sleep assessment tools while simultaneously evaluating both the duration of use and pre-sleep usage behaviors across different technological devices, such as televisions, tablets, and smartphones, remains limited. 8 Moreover, the evidence derived from large-scale studies conducted among primary school children remains inconclusive. 9 The present study aims to address these gaps by using a substantial sample and a multidimensional approach to examine the association between technology use patterns and sleep quality among primary school children.

The World Health Organization (WHO) and the American Academy of Pediatrics (AAP) have established age-based screen time limits. They recommend that elementary school-aged children (6-10 years old) should limit their screen time to 1 to 1.5 hours per day. 10 11 As indicated by the WHO, the duration of screen exposure should be limited to 2 hours per day for children and adolescents aged 5 to 17, as this has been identified as a significant risk factor with regard to cognitive and psychosocial development. 11

Current studies have shown that watching television and using tablets and smartphones for more than 2 to 3 hours a day is associated with decreased sleep quality, increased sleep problems, and an increased risk of obesity. Studies have also shown that such behavior is linked to attention deficit in early school years, poor academic performance, and attention deficit hyperactivity disorder (ADHD) in children. 8 12 13 14 The deleterious effects of uncontrolled technology use on sleep quality have been demonstrated to have an adverse effect on the growth and development processes of children. 7 15 Recent technological advances and the increasing amount of time spent by elementary school children in front of screens have made it clear that studies focusing on sleep quality are important for the protection and improvement of children's health from a public health perspective. 16 17

In the contemporary era, digital technologies have become an integral component of both the educational sphere and the quotidian lives of children of elementary school age. The present study explores the relationship between increased screen use for educational purposes at school and at home, and the use of television, tablets, and smartphones for entertainment, and their effect on children's screen exposure. This situation requires a detailed examination of the effects of technology use, especially during the hours preceding sleep, on the quality of sleep in elementary school children. 18 19

The objective of the current study is to examine the relationship between technology usage duration and characteristics, and sleep quality in elementary school children, a period when exposure to technology increases in terms of education and social development.

## METHODS

The present study constitutes a descriptive correlational study with the objective of determining the effect of technology use on sleep quality in elementary school-aged children. The study was conducted between January and May 2023 at an elementary school in the provincial centre, which serves as a learning hub for children hailing from diverse regions across Turkey. The student population of the school (N:1,352) was selected through convenience sampling, with consideration given to family and regional profiles. The study sample was divided into 4 strata for grades 1, 2, 3, and 4 of elementary education, and the sample size was calculated using the formula n = N t2pq / d2 (N-1) + t2pq, taking into account the number of target individuals in each age group in each grade, with a minimum target of 300 students (25% frequency, 95% C.I.). Approval was obtained from the developers of the data collection tool utilised in the study. Ethical committee approval (no. 0370/2023) and institutional permission from the school where the study was conducted were obtained.

Initially, all students enrolled in grades 1 through 4 at the selected elementary school were considered eligible for participation. The school administration was instrumental in the dissemination of study information forms and informed consent documents to parents. Participation in the study was voluntary, and only students whose parents provided written informed consent were included in the study. In order to ensure adequate representation across grade levels, the student population was stratified according to grades 1, 2, 3, and 4. The required sample size for each stratum was calculated proportionally based on the total number of students enrolled in each grade. A convenience sampling approach was employed in order to recruit students, with recruitment occurring within each stratum until the targeted sample size was reached. The data was collected using a combination of online and face-to-face methods. Following the screening of the data, 17 questionnaires were excluded on the basis that they contained incomplete or missing responses. Consequently, analyses were conducted with data obtained from 600 students who completed all study forms fully and correctly.

The study examined students' age, gender, grade level, technology use, and sleep patterns and durations. The data were collected using two instruments: a Descriptive Characteristics and Technology Use Questionnaire developed by the researchers and based on the relevant literature, and the Children's Sleep Habits Questionnaire (CSHQ). The descriptive questionnaire comprised items pertaining to sociodemographic characteristics (age, gender, grade level), daily technology use habits, the types of technological devices used (television, tablet, smartphone, computer), the duration of screen exposure, and pre-bedtime technology use behaviours. The questionnaire also included questions regarding sleep duration and bedtime routines. Prior to data collection, the content and clarity of the questionnaire items were evaluated by experts in paediatric nursing and public health.

The CSHQ, the most widely utilised instrument for the evaluation of children's sleep patterns, disorders, and quality, was employed in the current study. 20 The scale is a valid and sufficient measurement tool for the screening of sleep-related problems in school children, as demonstrated in a Turkish pediatric population. 21 The parent is responsible for completing the form in accordance with retrospective reporting and invited to evaluate their child's sleep patterns over the previous week, categorizing them into eight sleep domains. Each question item is scored on a 3-point scale across 33 questions. The sleep quality scale ranges from a minimum of 33 to a maximum of 99 points. Children who attain a total score of 41 or above on the CSHQ are categorized as having clinically-significant sleep problems according to the scale. The scale demonstrated an alpha coefficient of 0.796. 19

The data were analyzed using SPSS Statistics for Windows (IBM Corp.) version 25.0. Descriptive statistics, including percentage distributions and mean values, were utilized, and the Kolmogorov-Smirnov test was conducted to ascertain whether the data followed a normal distribution. The findings revealed deviations from the normal distribution, particularly among female participants, where skewness and kurtosis values exceeded acceptable thresholds. The Mann-Whitney U test was employed to compare the mean between two groups, and the Kruskal-Wallis χ ^2^ test was used to compare the mean between more than two groups. The objective of the present study was to assess the relationship between technology utilisation prior to bedtime and the quality of sleep. To this end, Spearman's rank correlation coefficient was utilized as a statistical instrument. The subsequent analysis was carried out using Dunn-Bonferroni tests. The prediction of children's sleep habits, as measured by the CSHQ, and their sociodemographic variables was evaluated using a multiple linear regression analysis. Group comparisons were performed using non-parametric tests, and a multiple linear regression analysis was conducted to determine independent predictors of CSHQ total scores. Statistically speaking, a *p* -value < 0.05 was deemed to be statistically significant. 22

## RESULTS

The mean age of the 600 elementary school children was 8.67 years (± 1.11), with 51.2% of the sample being girls (n = 307) and 48.8% being boys (n = 293). The mean score on the Children's Sleep Habits Questionnaire (CSHQ) was found to be 52.79 ± 6.73. The study revealed that 80.2% of the students had access to a tablet device at home, 98.7% had a television, 21.2% possessed a smartphone, and all children used at least one technological device. The average sleep duration was found to be 7.69 ± 1.02.

The demographic composition of the student body is as follows: 105 students (17.7%) are in 1st grade, 121 (20.2%) are in 2nd grade, 200 (33.3%) are in 3rd grade, and 174 (28.8%) are in 4th grade. An analysis of the routines engaged in by the children participating in the study prior to sleep revealed that 57.8% of the subjects engaged in toothbrushing, 32% consumed milk, 42.6% engaged in reading, 60.5% watched television, 53.5% engaged in computer, phone, or tablet use, 32.5% participated in sports or activities, and 52.8% engaged in academic study. The study revealed that 94.3% of children's families permit them to use electronic devices, 29.3% of children have electronic devices in their rooms, 80.2% have tablets at home, 98.7% have televisions at home, 99.7% have phones at home, 21.2% of children have their own smartphones, and 55% have their own tablets.

Table 1 shows a comparison of students' CSHQ total scoresaccording to various sociodemographic and technology-related variables. A non-significant disparity was identified between the mean CSHQ scores of girls and boys (girls: 52.66 ± 6.53; boys: 52.91 ± 6.93; U = 44384.5; *p* = 0.780). A statistically significant difference was identified when CSHQ scores were compared according to children's grade levels (χ ^2^ = 7.820; *p* = 0.049). The mean score observed in 3rd-grade students was the highest (53.68 ± 7.39), while the lowest mean score was observed in 2nd-grade students (51.53 ± 5.55). The mean score obtained by children who used technological devices (53.42 ± 6.38) was found to be significantly higher than that of children who did not use them (42.29 ± 2.24) (U = 30,000; *p* < 0.001). The mean CSHQ score of children who used technological devices before bedtime (54.56 ± 7.89) was found to be statistically significantly higher than that of children who did not use them (52.05 ± 6.04) (U = 29990.0; *p* < 0.001). A statistically significant difference was identified in the CAA scores according to television viewing time (χ ^2^ = 96.317; *p* < 0.001). The mean score of children who did not watch television (42.29 ± 2.24) was lower than that of children who watched ≤ 2 hours (53.34 ± 6.37) and > 2 hours (53.69 ± 6.44). A statistically significant relationship was identified between the duration of tablet usage and the resulting CSHQ scores (χ ^2^ = 96.317; *p* < 0.001). The mean score of children who did not use tablets (42.29 ± 2.24) was lower than that of children who used tablets for ≤ 2 hours and >2 hours. A statistically significant difference was identified between CSHQ scores and smartphone usage time (χ ^2^ = 100.386; *p* < 0.001). The mean score of children who did not use smartphones (42.29 ± 2.24) was lower than that of children who used them for ≤ 2 hours (53.27 ± 6.46) and > 2 hours (54.63 ± 5.56).

**Table 1:** Comparison of Children's Sleep Habits Questionnaire total scores according to sociodemographic and technology-related variables (N = 600)

Variable		n	Mean ± SD	Test statistic	*p* -value
**Gender**	Girls	307	52.66 ± 6.53	U = 44,384	0.780
	Boys	293	52.91 ± 6.93		
**Grade level**	1st grade	105	52.85 ± 6.33	χ ^2^ = 7.820	0.049
	2nd grade	121	51.53 ± 5.55		
	3rd grade	200	53.68 ± 7.39		
	4th grade	174	52.59 ± 6.83		
**Use of technological devices**	Yes	566	53.42 ± 6.38	U = 30.000	< 0.001
	No	34	42.29 ± 2.24		
**Technological device use at bedtime**	Yes	176	54.56 ± 7.89	U = 29,900	< 0.001
	No	424	52.05 ± 6.04		
**Television viewing time**	None	34	42.29 ± 2.24	χ ^2^ = 96.317	< 0.001
	≤ 2 h/day	439	53.34 ± 6.37		
	> 2 h/day	127	53.69 ± 6.44		
**Tablet use time**	None	34	42.29 ± 2.24	χ ^2^ = 96.317	< 0.001
	≤ 2 h/day	439	53.34 ± 6.37		
	> 2 h/day	127	53.69 ± 6.44		
**Smartphone use time**	None	34	42.29 ± 2.24	χ ^2^ = 100.386	< 0.001
	≤ 2 h/day	506	53.27 ± 6.46		
	> 2 h/day	60	54.63 ± 5.56		

Abbreviation: SD, standard deviation.

Notes: Higher Children's Sleep Habits Questionnaire scores indicate poorer sleep quality. Mann-Whitney U, Kruskal-Wallis χ ^2^ .

Table 2 shows the effect sizes and postoperative comparisons for variables that have been found to be significantly related to students' sleep quality. The total scores achieved by the CSHQ did not vary to a significant extent according to gender ( *p* = 0.780), suggesting that sleep habits are comparable between girls and boys. However, a statistically significant difference was observed across grade levels ( *p* = 0.049), although the effect size was small, suggesting limited practical significance. The utilization of technological devices by children was found to be significantly associated with elevated CSHQ scores when compared with the non-use of such devices ( *p* < 0.001). This finding indicates a substantial effect size, suggesting that children who use these devices experience poorer sleep quality. Conversely, the utilization of technological devices at bedtime was found to be associated with significantly poorer sleep outcomes ( *p* < 0.001).

**Table 2:** Effect Sizes and post-hoc comparisons for variables significantly associated with sleep quality

Variable	Comparison	Effect size	Interpretation
Technological device use	Yes vs No	r ≈ 0.58	Large effect
Technological device use at bedtime	Yes vs No	r ≈ 0.21	Small–moderate effect
Grade level	Overall	η ^2^ ≈ 0.013	Small effect
TV viewing time	None vs ≤ 2 h	η ^2^ ≈ 0.16	Moderate–large
TV viewing time	None vs > 2 h	η ^2^ ≈ 0.17	Moderate–large
Tablet use time	None vs ≤ 2 h	η ^2^ ≈ 0.16	Moderate–large
Smartphone use time	None vs > 2 h	η ^2^ ≈ 0.18	Large effect

Note: *Post hoc: Dunn-Bonferroni tests showed that children who did not use technological devices had significantly lower CSHQ scores compared to all device-using groups ( *p* < 0.001).

Television, tablet, and smartphone usage durations were all found to be significantly associated with CSHQ total scores (all *p* < 0.001). Subsequent post-hoc analyses revealed that children who did not engage with screen-based media exhibited significantly superior sleep quality compared to those using screens for ≤ 2 hours or > 2 hours per day. It is worth noting that smartphone use exceeding 2 hours per day demonstrated the largest effect size, thereby underscoring its strong association with impaired sleep habits ( Table 2 ).

Table 3 shows that technological device-related variables were identified as significant predictors of children's sleep habits in the multiple linear regression analysis. The overall model was statistically significant, explaining 42% of the variance in CSHQ total scores (R ^2^ = 0.41; *p* < 0.001). In the analysis of the available data, it was found that the strongest predictor of poorer sleep quality was smartphone use for more than 2 hours per day (β = 0.34; *p* < 0.001). Furthermore, general use of technological devices (β = 0.31; *p* < 0.001), tablet use for more than 2 hours per day (β = 0.23; *p* < 0.001), prolonged television wacthing (β = 0.21; *p* < 0.001), and the presence of a technological device in the child's bedroom (β = 0.18; *p* < 0.001) were independently associated with higher CSHQ scores. As demonstrated in Table 3 , the strongest independent predictor of poor sleep habits among children was identified as the use of smartphones for more than 2 hours per day.

**Table 3:** Multiple linear regression analysis predicting Children's Sleep Habits Questionnaire total score

Predictor variables	β (standardized)	SE	t	*p* -value
Gender (boy)	0.03	0.41	0.74	0.460
Grade level	0.07	0.18	1.92	0.055
Use of technological devices (yes)	0.31	0.72	7.85	< 0.001
Technological device in bedroom (yes)	0.18	0.39	4.62	< 0.001
TV viewing time (> 2 h/day)	0.21	0.44	5.10	< 0.001
Tablet use time (> 2 h/day)	0.23	0.46	5.64	< 0.001
Smartphone use time (> 2 h/day)	0.34	0.51	8.92	< 0.001

## DISCUSSION

The present study examined the relationship between technology use and sleep habits among elementary school-aged children and identified key predictors of poor sleep quality. The findings demonstrate that screen-based behaviours are strongly associated with impaired sleep habits, with smartphone use exceeding 2 hours per day emerging as the most powerful independent predictor of higher CSHQ total scores. This finding is consistent with the existing body of evidence that suggests increased use of technological devices may pose a significant risk to sleep health in children.

Specifically, the analysis revealed that smartphone usage exceeding 2 hours per day emerged as the most robust independent predictor of high CSHQ scores, suggesting poorer sleep quality. It has been demonstrated that children who utilize technological devices for a minimum of 2 hours per day are significantly more likely to exhibit disrupted sleep habits. These findings are consistent with previous research demonstrating that increased exposure to digital media has a detrimental effect on the sleep health of children. Chandranaik et al. 23 reported that time spent with technology has a detrimental effect on the quality of sleep among school-aged children. Similarly, Chaput et al. 24 found that greater screen time was positively associated with reduced sleep efficiency in children. Özsavran et al. 25 also reported a significant increase in sleep problems associated with problematic media use in elementary school-aged children. Unlike many previous studies that primarily focused on adolescents or examined total screen time alone, the present study concentrated on a large sample of elementary school children and evaluated multiple device types as well as timing of use. It is important to note that the identification of smartphone use for more than 2 hours per day, particularly prior to bedtime, as an independent predictor of poor sleep quality constitutes a significant contribution to the existing literature on the subject.

The mean age of participants was 8.67 ± 1.11 years, and almost all children reported using technological devices. This extensive exposure, in conjunction with the substantial effect size observed, indicates that technology use functions as a considerable risk factor for diminished sleep quality in this age group. It has been demonstrated that children who utilize technological devices prior to bedtime exhibit significantly worse sleep quality in comparison to those who do not engage with such devices. In accordance with the findings of preceding research, both general screen exposure and bedtime technology use, particularly exposure to blue light, have been demonstrated to disrupt sleep and potentially impede the healthy development of children. 8 23 26 27 28

Environmental factors, such as exposure to technological devices, have been demonstrated to exacerbate sleep-related problems, including delayed sleep onset, reduced sleep efficiency, and increased physiological arousal. It has been demonstrated that sleep disturbances can have a detrimental effect on children's physical maturation, neurocognitive development, emotional regulation, and behavioural adaptation. 26 Furthermore, insufficient sleep and impaired sleep quality have been associated with deficits in attention, intelligence, and academic performance in children and adolescents. 29

In accordance with preceding studies, no substantial gender disparities were identified in overall sleep patterns, indicating that technology-related sleep disturbances affect both male and female subjects in a comparable manner. 23 25 26 30 While a statistically significant correlation was observed between grade level and sleep habits, the effect size was negligible, suggesting that demographic and school-related factors account for only a modest proportion of the variability in sleep outcomes. These findings are consistent with the literature on pediatric sleep, which demonstrates that statistically significant demographic effects frequently possess limited clinical relevance. 2 23 27 31

In contrast, technology-related variables, particularly device type and duration of use, demonstrated larger effect sizes, thereby underscoring their importance in the multifactorial etiology of childhood sleep problems. 2 8 15 27 The strongest association with disrupted sleep habits was observed among individuals who used their smartphones for more than 2 hours per day. Smartphones offer a plethora of stimulating and interactive content, which is frequently utilized in the evening. This has the potential to delay bedtime and elevate cognitive and emotional arousal. Furthermore, exposure to blue light emitted from electronic devices has been demonstrated to suppress melatonin secretion, thereby disrupting the circadian rhythms of children. 2 9 26 32 33 34 In addition to technology-related exposure, sleep duration itself may represent an important factor influencing overall sleep quality outcomes. Although the present study evaluated average sleep duration, detailed circadian timing variables such as bedtime and wake-up time were not assessed. Consequently, it can be hypothesized that reduced sleep duration may partially contribute to elevated CSHQ scores, regardless of device use behaviors. This may act as a potential confounding factor in the observed associations. It has been demonstrated in previous research conducted in the field of paediatric sleep science that a shorter duration of sleep, in addition to irregular sleep schedules, has been found to be independently associated with a poorer quality of sleep and the presence of behavioral sleep problems in children. Further research is required to incorporate detailed sleep timing characteristics and longitudinal assessment designs. This will assist in clarifying the complex interplay between technology exposure, sleep duration, circadian patterns, and sleep quality.

In accordance with international guidelines, children who did not use screens or who limited their screen exposure to ≤ 2 hours per day demonstrated significantly better sleep quality compared with those exceeding this threshold. These results align with recommendations from the American Academy of Pediatrics and the World Health Organization, emphasising excessive smartphone use as a key, modifiable target for both clinical counselling and public health interventions aimed at improving paediatric sleep health. 10 11 Routine screening of screen time behaviors, particularly during school health assessments, may facilitate early identification of children at risk.

The present study boasts numerous strengths, thereby enhancing the robustness and validity of its findings. Firstly, the relatively large sample size of elementary school-aged children provided sufficient statistical power. While most of the exisiting studies have focused on adolescents, the curret research highlights the impact of technology use on sleep health at an earlier developmental stage, emphasising the need for early preventive interventions. Secondly, sleep habits were assessed using the CSHQ, a widely validated and commonly used instrument in pediatric sleep research. Thirdly, the study examined multiple dimensions of technology use, including smartphones, tablets, and television viewing, as well as device use before bedtime, rather than focusing solely on total screen time. Finally, the employment of multivariable regression analysis enabled the control of potential confounding factors, thereby strengthening the inference that technology-related behaviours independently contribute to sleep problems.

Several limitations should also be acknowledged. The cross-sectional design precludes causal inference, and reliance on parent-reported data may introduce recall or social desirability bias. It is evident that the incorporation of objective sleep measures, sleep timing variables (e.g., bedtime and wake-up time), and contextual behavioral factors into future longitudinal studies will facilitate the elucidation of causal pathways.

In conclusion, the present study demonstrates that there is a strong correlation between the use of screen-based technology and the presence of poor sleep habits in elementary school-aged children. Smartphone use for more than 2 hours per day has been identified as the most powerful independent predictor of impaired sleep quality. Beyond the overall daily exposure, bedtime technology use has been demonstrated to contribute to adverse sleep outcomes, underscoring the significance of both behavioral and environmental factors. It is submitted that addressing excessive smartphone use in accordance with the guidelines established by the AAP and the WHO may play a critical role in promoting healthy sleep and overall child wellbeing. It is imperative that families, schools, and primary healthcare services implement routine assessment and regulation of screen exposure as a pragmatic, cost-effective approach to safeguard and enhance the sleep health of children.
